# Fenton Treatment of Filter Backwash Water for Recycling in DWTP

**DOI:** 10.1002/wer.70337

**Published:** 2026-03-13

**Authors:** Luana Valverde Pereira, Matheus Carlos de Sá, Isabella Pereira de Lima, Lucas Coelho Rossi, Taciane de Oliveira Gomes de Assunção, Edgard Henrique Oliveira Dias, Sue Ellen Costa Bottrel, Renata de Oliveira Pereira

**Affiliations:** ^1^ Postgraduate Programme in Civil Engineering (PEC) Federal University of Juiz de Fora (UFJF), Faculty of Engineering Juiz de Fora Minas Gerais Brazil; ^2^ Department of Sanitary and Environmental Engineering (ESA) Federal University of Juiz de Fora (UFJF), Faculty of Engineering Juiz de Fora Minas Gerais Brazil

**Keywords:** advanced oxidative processes (AOP), aerobic endospores (AE), *Escherichia coli* (EC), protozoa, water reuse

## Abstract

Drinking water treatment plants (DWTPs) generate residues, such as filter backwash water (FBW), that can harm the aquatic environments if discharged into water sources. In this sense, FBW recycling has gained attention. Nevertheless, it requires treatment to maintain operational efficiency and water quality in the DWTP. This work analyzed the main variables interfering with Fenton treatment of FBW from a full‐scale DWTP aiming at removing turbidity and microorganisms. Bench‐scale tests successfully inactivated 100% of *Escherichia coli* . Desired removal of other parameters (99.1% of turbidity, 99.9% of color, and achieving a 2.5 log_10_ reduction of aerobic endospores) occurred during the sedimentation stage. There was a strong influence of the pH sample and the concentration of added hydrogen peroxide on the removal of microorganisms. This study represents an initial stage that should be continued to enable the removal of resistant microorganisms through oxidation by the Fenton reaction in FBW treatment.

## Summary

- Treatment of filter backwash water (FBW) enables safe water recycling in DWTP.
- Fe from FBW was used to promote Fenton reactions, minimizing reagent costs.
- AOP/Fenton was shown to be a potential process to inactivate microorganisms in FBW.

## Graphical abstract

The graphical abstract illustrates the treatment and recycling pathway of filter backwash water (FBW) generated in drinking water treatment plants. The proposed treatment innovatively applies the Fenton advanced oxidation process, using the iron naturally present in the FBW, with a sedimentation step for effective clarification.

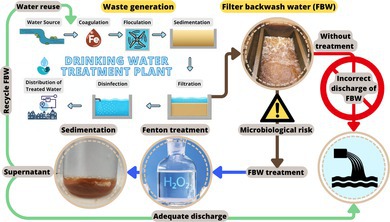

## 1 Introduction

The availability of safe drinking water has become one of the major challenges of the 21st century, given its fundamental importance for public health and societal development (Fouad et al. 2017). In this context, drinking water treatment plants (DWTPs) aim to ensure that quality water reaches the public water supply system for human consumption. One system used worldwide, including Brazil, is the conventional water treatment process, which involves coagulation, flocculation, sedimentation, filtration, and disinfection (Ćurko et al. 2013; Othman et al. 2021). These stages are intended to meet the potability requirements set forth by the Brazilian Drinking Water Quality Standards (Brasil 2021).

Inherent to the water treatment process, substantial quantities of waste with pollutant potential are generated in DWTP (Fouad et al. 2017). The cleaning of sedimentation tanks produces DWTP sludge, whereas the backwashing of filters generates filter backwash water (FBW). The properties of these by‐products result from the combination of the raw water properties, the adopted treatment technology, the added chemicals (e.g., coagulants), and their dosages (Ćurko et al. 2013; Mahdavi et al. 2017; USEPA 2002; Yang et al. 2006).

Filter backwashing stands out as the main source of residues in terms of volume produced at short intervals. The volume of FBW produced in a DWTP corresponds to approximately 2% to 10% of the total volume of treated potable water produced at the DWTP (Hu et al. 2021; Qian et al. 2020; USEPA 2002; Wolska and Urbańska‐Kozłowska 2023; Zielina and Dąbrowski 2021). It is estimated that, in Brazil, approximately 56.4% of the waste from DWTP is discharged into water sources without any treatment (IBGE 2017). Improper discharge of FBW into water bodies contributes to various environmental impacts, as the residue may contain heavy metals and pathogenic microorganisms and leads to the increasing concentration of solids and turbidity of surface waters (Gottfried et al. 2008; Mahdavi et al. 2018; Nasser et al. 2002). Such factors pose risks to aquatic life and public health (Gavlak et al. 2021; Silva and Sabogal‐Paz 2020). Therefore, adequate management of FBW becomes crucial, encompassing minimization, utilization, treatment, and adequate disposal of these residues.

As a result of the high volume of FBW produced in DWTP and the environmental impact of their inadequate discharge into water bodies, interest in the practice of recycling FBW in DWTP has gained prominence (Ismail 2020; Mady et al. 2023; Wolska and Urbańska‐Kozłowska 2023). However, research on the subject is still limited in the Brazilian scenario. The option to recycle FBW helps reduce the discharge of these residues into the environment, promotes water savings, and reduces chemical consumption (Arora et al. 2001; Gottfried et al. 2008; Mazari and Abdessemed 2020). However, direct recycling of FBW to the beginning of the water treatment process can introduce elements such as iron, manganese, trihalomethane precursors, and microorganisms that may be present in this residue (Arora et al. 2001; Bourgeois et al. 2004; Hu et al. 2021; Walsh et al. 2008). In addition, as filtration is the main step to physically remove protozoa (e.g., *Giardia* sp. cysts and *Cryptosporidium* sp. oocysts) in DWTP, the recycling of FBW could lead to the reintroduction of these microorganisms into the systems as FBW contains high protozoa concentrations (Gottfried et al. 2008; Loret et al. 2013; USEPA 2002; Wolska et al. 2024).

In the USA, the USEPA (2002) establishes technical guidance for filter backwash recycling. The goal is to ensure that FBW recycling practices do not compromise the effectiveness of the DWTP. However, the document does not present treatment criteria or quality standards for treated FBW. The main consideration of the USEPA's manual is the need to enhance pathogen control regarding *Cryptosporidium* sp. as FBW presents high levels of protozoa (USEPA 2002). Additionally, it recommends reintroducing FBW at a stage before coagulation, as recycling after the point of coagulant addition can disrupt the chemistry processes and impair treatment performance (USEPA 2002). Currently, there are no guidelines or regulations for the treatment and recycling of FBW in DWTP in Brazil.

Advanced oxidative processes (AOP) stand out due to their high efficiency in degrading organic compounds and relatively low operational cost. These processes are based on producing free radicals, such as hydroxyl radicals (HO•), which can nonselectively oxidize numerous organic contaminants or recalcitrant compounds into inorganic substances like carbon dioxide and water. When optimal conditions are used, it promotes the mineralization of contaminants (Covinich et al. 2014; Pandis et al. 2022). Furthermore, AOP can inactivate microorganisms (Covinich et al. 2014; Zhang et al. 2020).

AOP based on the Fenton reagent (AOP/Fenton) has been the subject of extensive research in recent years, recognized as an effective, versatile, dependable, and low‐cost technique among various AOP (Aldershof et al. 1997; Moresco et al. 2021; Tarr 2003). The hydroxyl (HO•) and perhydroxyl (HO_2_•) radicals are formed during the decomposition of hydrogen peroxide (H_2_O_2_) in the presence of iron (Fe^2+^) (Aldershof et al. 1997; Moresco et al. 2021; Tarr 2003). According to Aldershof et al. (1997), the main parameters controlling this reaction are well defined: hydrogen peroxide concentration, temperature, time, pH, and catalyst concentration. It is also possible to promote the formation of oxidants in a “Fenton‐like” process, in which Fe^3+^ and H_2_O_2_ are used as initial or intermediate reactants. The ability of the Fenton reactant (Fe^2+^/H_2_O_2_) to inactivate microorganisms is associated with its potential to generate reactive oxygen species, such as hydroxyl radicals (HO•) and superoxide (O_2_•^−^), as these radicals have the property of causing damage to microbial deoxyribonucleic acid (DNA) and cell membrane components (Schurman 2001).

Given the absence of specific regulations for FBW recycling in Brazil and taking into account the USEPA guidelines regarding this topic (USEPA 2002), the primary aim of this study was to evaluate the simultaneous removal of microorganisms and turbidity from FBW using a bench‐scale AOP/Fenton process. The study advances current understanding through two main innovations: (i) the assessment of the treatment efficacy regarding the removal of microorganisms, including aerobic endospores (AE) as indicators of protozoa; and (ii) the use of the intrinsic iron naturally present in the FBW as a catalyst for the Fenton reaction.

## 2 Methods

### 2.1 Sample Collection and Characterization

FBW samples were collected from a drinking water treatment plant located in Minas Gerais State, Southeast Brazil. The DWTP comprises a conventional treatment system (i.e., coagulation, flocculation, sedimentation, filtration, and chlorine disinfection), operates with an average flow of 620 L·s^−1^, and uses iron‐based coagulants (FeCl_3_ and Fe_2_(SO_4_)_3_). The filters are backwashed daily using treated water from the DWTP itself. Each of the 12 sand filters requires approximately 200 m^3^ of treated water for backwashing, resulting in a total daily consumption of around 2400 m^3^ for the complete backwashing process.

Three FBW samples were collected on November 7th, 21st, and 28th, 2023. Each sample was composed of five aliquots of approximately 5.0 L collected every 2 min during the backwash process of an arbitrary filter in the DWTP, totalling a backwash time of about 10 min. FBW samples were characterized in terms of physicochemical and microbiological parameters as presented in Table 1. Before the quantification of *Escherichia coli* (EC) and aerobic endospores (AE), an elution step was performed aiming to detach the microorganisms from the solids within the raw samples (UKEA 2003).

**TABLE 1:** Physicochemical and microbiological parameters and methodologies for characterizing the FBW.

Microbiological analysis	Method	Methodology	References
*Escherichia coli* (EC)	Membrane filtration and Colilert	BS EN ISO 9308‐1—Detection and enumeration of *E. coli* and Coliform	(BSI 2009)
Aerobic endospores (AE)	Membrane filtration	SMEWW n° 9218‐B—Aerobic endospores	(APHA et al. 2017)

Physicochemical analysis	Method	Methodology	References
pH	Potentiometric	SMEWW n° 4500—(H^+^) pH	(APHA et al. 2017)
True color, apparent color	Colorimetric	SMEWW n° 2120—Color	
Turbidity	Nephelometric	SMEWW n° 2130—Turbidity	
COD	Colorimetric	SMEWW n° 5220—Chemical oxygen demand	
Total Fe, dissolved Fe, Fe II, Fe III	Colorimetric	SMEWW n° 3500—(Fe) Iron	
TS, TFS, TVS, TSS	Gravimetric	SMEWW n° 2540—Solids	
TOC	Combustion/infrared	SMEWW 5310	
Peroxide residual	Iodometric titration	PT‐H_2_O_2_	(Sawyer et al. 1994)

Abbreviations: COD, chemical oxygen demand; FBW, filter backwash water; Fe, iron; SMEWW, Standard Methods for the Examination of Water and Wastewater; TFS, total fixed solids; TOC, total organic carbon; TS, total solids; TSS, total suspended solids; TVS, total volatile solids.

The microbiological analyses aimed to evaluate the main microbial indicators found in the DWTP residues. EC is a widely recognized bacterial indicator of fecal contamination (Petris et al. 2019). AE share important characteristics with *Giardia* sp. cysts and *Cryptosporidium* sp. oocysts in terms of their removal through clarification processes in water treatment, and stand out for being more numerous in raw waters and for being more practical and accessible for monitoring (Mazoua and Chauveheid 2005; Oliveira et al. 2018).

### 2.2 Experimental Design for Optimization

Following the characterization of FBW samples, bench‐scale tests of the Fenton reaction were conducted to determine the optimal operational parameters of the FBW Fenton treatment in order to remove suspended solids (turbidity) and microorganisms (EC and AE). The experimental design was based on factorial analysis (2^k^), in which the experiment simultaneously evaluates the effects of “k” variables that significantly influence the process (Montgomery 2017). Two experimental designs were performed. In the first one, three factors were analyzed (*k* = 3): (i) pH; (ii) H_2_O_2_:Fe ratio; and (iii) reaction time. In the second experiment, based on the results of the first design, two factors were analyzed (*k* = 2): (i) pH; and (ii) H_2_O_2_:Fe ratio. In both experimental designs, for each factor, two levels (lower and upper) were considered, as well as the central point (average between the lower and the upper levels). In all cases, tests were performed in duplicate using 500 mL of FBW samples. After the Fenton treatment, taking into account the reaction and sedimentation times, aliquots were collected for the analyses of turbidity, residual H_2_O_2_, and indicator microorganisms. It is noteworthy that analyzing residual peroxide allows for quantifying the consumption of H_2_O_2_ and verifying the occurrence of the reaction. Aliquots for microbiological analyses received 0.01 mL of 10% (m/v) sodium bisulfite (Control Lab Ltda., Pinhais, Paraná, Brazil) per 1.0 mL of the sample aiming to halt the Fenton reaction.

The evaluation of the results of the factorial experiments was performed using the Minitab statistical software (Pennsylvania State University, USA), version 22.2.2 (Minitab 2024), at 95% confidence level. Pareto charts allow the assessment of which factors are most relevant (pH; H_2_O_2_:Fe ratio; reaction time) for the response variables of the study (turbidity; EC; AE). The magnitude of each effect is represented by the bars, whereas the horizontal line indicates the *p*‐value. The columns that cross this line show statistically significant influence on the results. Contour plots provide an integrated visualization of the interaction between the factors and the response variables, with the axes covering the range of values used in the experiments. The FBW samples were characterized on the same day they were collected, whereas the Fenton treatment was carried out on the following day.

**FIGURE 1:**
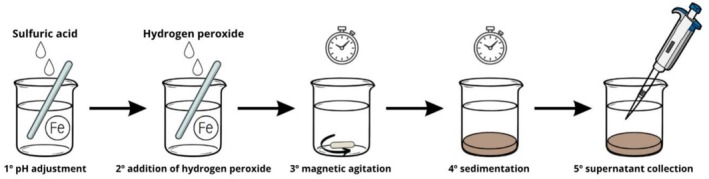
Scheme of Fenton treatment steps of the filter backwash water (FBW).

The experimental procedures were conducted following the steps of the Fenton treatment, as illustrated in Figure 1. Initially, the pH was adjusted to the desired value using concentrated sulfuric acid (NEON, Suzano, São Paulo, Brazil). Next, the necessary volume of hydrogen peroxide at 35% (Química Moderna, Santana de Parnaíba, São Paulo, Brazil) was added to achieve the desired H_2_O_2_:Fe ratio. The sample was then subjected to agitation by magnetic stirrers (IKA C‐MAG HS 7, Campinas, São Paulo, Brazil) for a predetermined time interval for the oxidation reaction, followed by a chosen resting period for sedimentation. In the present study, the Fenton reaction was conducted under ambient temperature (≈20°C), reflecting realistic conditions for Brazilian DWTP applications. Finally, the supernatant was collected for the assessment of the parameters of interest.

### 2.3 Experimental Design 1

**FIGURE 2:**
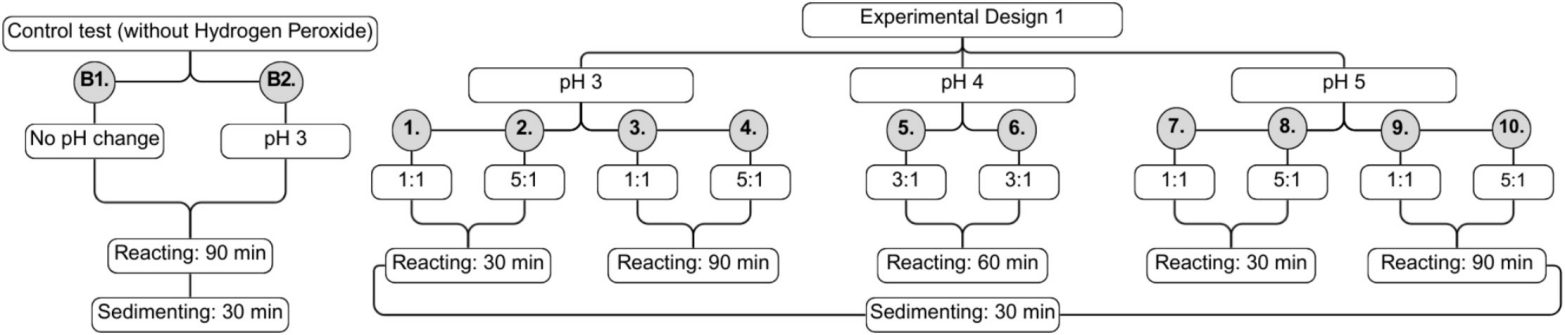
Flowchart of the 2^3^ factorial experimental design 1 (FED1).

The first 2^3^ factorial experimental design presented the following lower level, central point (average between lower and upper levels), and upper level, respectively: 3.0, 4.0, and 5.0 for pH; 1:1, 3:1, and 5:1 for H_2_O_2_:Fe ratio; and 30, 60, and 90 min for reaction time. The statistical treatment of the data was performed using the remaining turbidity and the removal rate (%) of AE. Figure 2 illustrates the Factorial Experimental Design 1 (FED1) variables of each assay (1 to 10), as well as the analytical blanks (B1 and B2). Assay B1 consisted of the sample without pH alteration and without the addition of H_2_O_2_, whereas B2 was conducted at pH 3 and without the addition of H_2_O_2_. These control assays were performed to verify the variations that occurred when the Fenton treatment was not applied under the same experimental conditions.

### 2.4 Experimental Design 2

**FIGURE 3:**
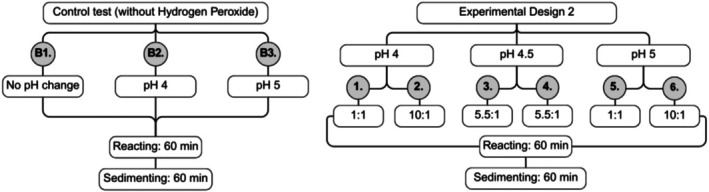
Flowchart of the 2^2^ factorial experimental design 2 (FED2).

Considering the optimal results obtained from the first factorial analysis, new specifications were investigated for the second 2^2^ factorial experiment. The second 2^2^ factorial experiment design presented the following lower level, central point (average between lower and upper levels), and upper level, respectively: 4.0, 4.5, and 5.0 for pH; and 1:1, 5.5:1, and 10:1 for H_2_O_2_:Fe ratio. The pH range was restricted to 4.0–5.0 based on the best values found to achieve the lowest turbidity and the best removal rates (%) of AE and EC. Variations in H_2_O_2_ concentration were increased to achieve higher removal rates of AE while maintaining the best H_2_O_2_ concentration conditions to achieve lower turbidity. In all cases, reaction and sedimentation times were fixed at 60 min. The flowchart of the Factorial Experimental Design 2 (FED2) with the steps of each assay (1 to 6) performed, as well as the analytical blanks (B1, B2, and B3), is shown in Figure 3. Assay B3 consisted of the sample without the addition of H_2_O_2_ and at pH 5.0.

### 2.5 Final Fenton Treatment

**FIGURE 4:**
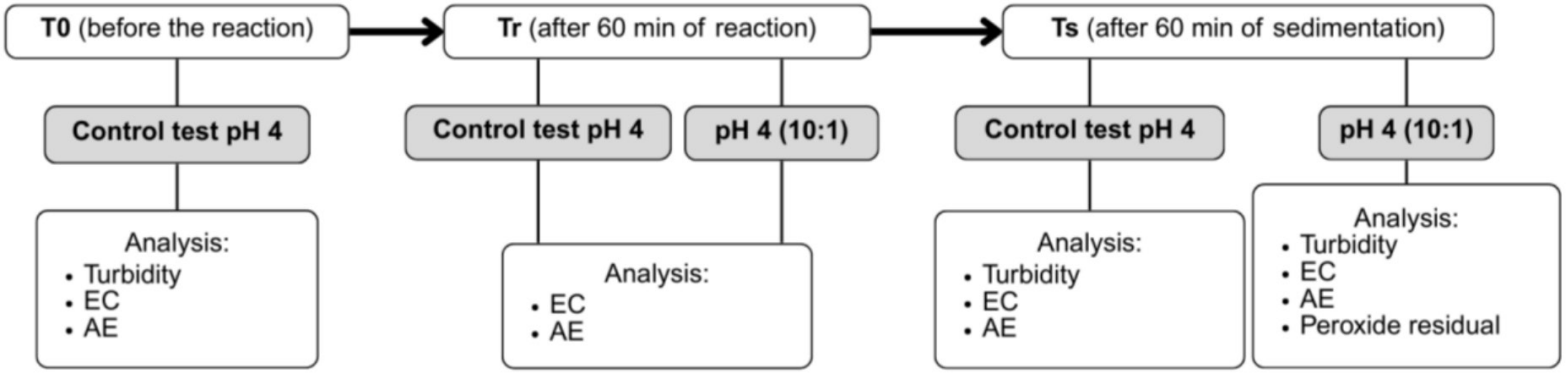
Flowchart of optimal Fenton treatment. AE, aerobic endospores; EC, *Escherichia coli*; T0, initial time, before the reaction; Tr, reaction time; Ts, sedimentation time.

Concluding the experiments for this FBW treatment research, a final assay was conducted with the optimal conditions in terms of pH (4.0) and H_2_O_2_:Fe ratio (10:1) obtained from the FED2, assessed regarding the removal rate (%) of AE. A flowchart representing the final Fenton treatment is shown in Figure 4. The assessment of optimal conditions for FBW treatment was primarily aimed at optimizing microbiological removal. Previous stages of the experiment focused on AE removal as the main optimization parameter, aligning with the safety guidelines outlined by the USEPA (2002) for FBW recycling. However, it is important to note that other parameters such as turbidity and EC assessment were also considered to ensure the overall safety of the process. This strategic approach aims to provide a comprehensive understanding of the impact of ideal treatment conditions, contributing to a more refined and effective planning of the overall FBW treatment process.

## 3 Results and Discussion

### 3.1 Characterization of FBW Samples

Table 2 presents the characterization of FBW samples in terms of physicochemical and microbiological parameters. It was noted that the results of some parameters analyzed ranged considerably. This is largely because these are real samples, subject to variations in terms of quality of the raw water, quantity of coagulation chemicals used, filter backwashing operation, and sampling (Brazão et al. 2021).

**TABLE 2:** Physicochemical and microbiological results of the FBW characterization.

Analysis	FBW collection 1 11/07/2023	FBW collection 2 11/21/2023	FBW collection 3 11/28/2023	Average	Standard deviation
EC (log_10_ cfu.100 mL^−1^)	2.8	3.1	2.5	2.8	0.3
AE (log_10_ cfu.100 mL^−1^)	3.8	4.0	3.8	3.9	0.1
pH	7.0	6.8	6.7	6.9	0.2
Turbidity (NTU)	70.0	89.7	57.1	72.3	16
True Color (Hu)	18.8	13.1	8.8	13.6	5
Apparent Color (Hu)	683	855	595	711	132
TOC (mg·L^−1^)	—	8.2	9.4	8.8	
TS (mg·L^−1^)	179	173	163	172	8
TVS (mg·L^−1^)	53.0	40.8	54.6	49.5	8
TSS (mg·L^−1^)	76.0	146.0	188.0	136.7	57
VSS (mg·L^−1^)	20.0	17.7	63.4	33.7	26
COD (mg·L^−1^)	14.0	37.0	90.0	47.0	39
Total Fe (mg·L^−1^)	44.0	44.9	18.3	35.7	15
Dissolved Fe (mg·L^−1^)	7.0	2.6	—	4.8	3
Fe ll (mg·L^−1^)	14.8	22.6	—	18.7	6
Fe lll (mg·L^−1^)	29.2	22.3	—	25.8	5

Abbreviations: AE, aerobic endospores; COD, chemical oxygen demand; EC, *Escherichia coli*; FBW, filter backwash water; Fe, iron; TS, total solids; TSS, total suspended solids; TVS, total volatile solids; VSS, volatile suspended solids.

When compared to the literature, the observed turbidity values (57.1–89.7 NTU) fall within the expected range of 58.0–171.0 NTU reported by Di Bernardo and Dantas (2005), who studied FBW in four DWTP in São Paulo State (Brazil), including those with sand filtration systems. Wolska and Urbańska‐Kozłowska (2023) showed results ranging from 7.8 to 92.0 NTU in a study carried out in Poland, in a DWTP using sand filters. The same applies to apparent color, with values observed in this study (595–855 Hu) compatible with the range reported in the Brazilian literature for sand filters: 290–4990 Hu (Campos 2014); and 200–2690 Hu (Di Bernardo and Dantas 2005). For true color, the values observed in this study (8.8–18.8 Hu) are similar to those reported by Wolska and Urbańska‐Kozłowska (2023), which ranged from 6.0 to 11.0 Hu.

During the study, which was conducted in the rainy season, the turbidity of surface waters tended to increase because solids (soil) are washed into surface waters such as rivers and lakes. This leads to an increase in the amount of coagulant required in DWTP and, consequently, a greater retention of solids in filters and an increase in turbidity of the FBW. However, the turbidity values of the FBW samples tested did not exceed 100 NTU.

For total solids (TS), the results obtained (163–179 mg·L^−1^) are close to the range values of 80–367 mg·L^−1^ reported in the literature (Di Bernardo and Dantas 2005). For suspended solids (SS), the values found in this work were 41–55 mg·L^−1^, whereas concentrations of 20–210 mg·L^−1^ were reported in the literature (Di Bernardo et al. 2002). A study in a DWTP in Egypt, which also uses sand filters, showed values between 160 and 249 mg·L^−1^ for SS (Abdel‐Shafy et al. 2018).

The values of chemical oxygen demand (COD) ranged from 14 to 90 mg. L^−1^, whereas values in the literature ranged from 30 to 50 mg. L^−1^ (Di Bernardo et al. 2002). Other studies in Brazilian DWTP that use sand filters and aluminum‐based coagulants found values of 63 mg·L^−1^ (Petris et al. 2019) and 132 mg·L^−1^ (Gavlak et al. 2021). According to Silva et al. (2021), the analysis of COD is susceptible to overestimated results, considering the interference of metals present in samples. Considering the use of iron‐based coagulants in the DWTP, this interference probably is high since the metal was quantified in the FBW samples. The average of the total organic carbon (TOC) concentration was 8.8 mg·L^−1^. This value is close to values reported by Wolska and Urbańska‐Kozłowska (2023), who obtained TOC between 3.77 and 8.93 mgC·L^−1^ in a sand filter analyzed in a DWTP in Poland.

Total iron concentrations ranged from 18.3 to 44.9 mg L^−1^, with some values higher than those reported by Campos (2014), which ranged between 9.4 and 33.7 mg·L^−1^, by Petris et al. (2019), which reported 16.8 mg·L^−1^, and by Gavlak et al. (2021), which reported 19.8 mg. L^−1^. But all these DWTPs used Polychloride Aluminum (PCA) as coagulant. It is worth mentioning that the Brazilian standards for effluent discharge into water bodies require levels of dissolved iron below 4.0 mg. L^−1^ (Brasil 2011). Nevertheless, in the first sample, the concentration exceeded this value. High levels of iron can degrade the aquatic environment, as iron can precipitate out of solution and accumulate in sediments, affecting benthic organisms and altering habitat structure as reported by Jia et al. (2018). It can also form silt banks and promote water siltation (Gavlak et al. 2021). On the other hand, the high iron concentrations in the FBW enable the Fenton process treatment before recycling, reducing requirements for external Fe sources and favoring sustainable management of DWTP waste.

*E. coli* results found in this study ranged from 2.5 to 3.1 log_10_ cfu.100 mL^−1^. Ismail (2020) found values between 2.4 log_10_ cfu.100 mL^−1^ and 2.8 log_10_ cfu.100 mL^−1^ in two different treatment plants using sand filters in São Paulo State (Brazil). A similar result was shown by Wolska and Urbańska‐Kozłowska (2023), who found 2.6 log_10_ cfu.100 mL^−1^. Di Bernardo and Dantas (2005) reported EC concentrations ranging from 0.5 to 4.3 log_10_ MPN.100 mL^−1^.

Regarding AE, the levels in FBW samples ranged from 3.8 to 4.0 log_10_ cfu.100 mL^−1^. Values between 2.6 and 3.9 log_10_ cfu.100 mL^−1^ were found for AE analysis in Brazilian raw water by Oliveira et al. (2018). So far, to the best of our knowledge, no data on AE in FBW have been identified in the literature.

Based on the characterization of FBW samples performed in this study, its direct recycling to the beginning of the DWTP may not be advisable if no treatment is applied to the FBW. The properties of the FBW can impair the entire treatment process as a consequence of the high loads of solids and microorganisms. Therefore, the use of AOP/Fenton treatment systems to reduce such loads was tested, and the results are presented in the following sections.

### 3.2 Experimental Design 1

The results obtained from the 2^3^ factorial experimental design 1 (FED1) are presented in Table 3. Preliminary results showed that Fenton treatment achieved turbidity removal ranging from 88% to 95.4%. Best values at the central point were achieved in test ID 5 and 6 (pH = 4; initial H_2_O_2_ = 91.0 mg. L^−1^, reaction time = 60 min) with 95.4% and 95.0% turbidity removal, respectively. For AE, Fenton treatment showed removals ranging from 0.9 to 1.3 log_10_, with best results in test ID 7 (pH = 5; initial H_2_O_2_ = 30.0 mg. L^−1^; reaction time = 30 min) and 8 (pH = 5; initial H_2_O_2_ = 151.0 mg. L^−1^; reaction time = 30 min), both with 1.3 log_10_ removal. Although Fe solubility is favored at more acidic pH and can lead to faster reactions (Nogueira et al. 2007), in this experiment it was observed that the experiments at pH equal to 3 (test ID 1–4) did not provide additional gains in turbidity or AE removal compared to the other pH values tested. It is worth noting that in all samples to which peroxide was added, residual peroxide was also detected, indicating that H_2_O_2_ was not completely consumed in the reactions.

**TABLE 3:** Concentrations and removal rates of turbidity and AE obtained from the 2^3^ Factorial Experimental Design 1 (FED1): pH, initial H_2_O_2_ (mg·L^−1^), and reaction time (min) as factors.

Test ID	pH	Initial H_2_O_2_ (mg·L^−1^)	Reaction time (min)	Turb.(NTU)	Turb. rem. (%)	AE(log_10_ cfu. 100 mL^−1^)	AE rem. (%)	AE rem. (log)	Peroxide residual
Raw FBW	7	−	0	90	−	3.8	−	−	−
B1	7^a^	−	90	8.5	90.6	2.9	87.4	0.9	−
B2	3	−	90	7.2	92.0	3.0	84.2	0.8	−
1	3	30.0 (1:1)	30	6.4	92.9	2.9	87.4	0.9	+
2	3	151.0 (5:1)	30	8.0	91.1	2.9	87.4	0.9	+
3	3	30.0 (1:1)	90	10.7	88.1	2.9	87.4	0.9	+
4	3	151.0 (5:1)	90	5.3	94.1	2.8	90.0	1.0	+
5	4	91.0 (3:1)	60	4.1	95.4	2.8	90.0	1.0	+
6	4	91.0 (3:1)	60	4.5	95.0	2.6	93.7	1.2	+
7	5	30.0 (1:1)	30	5.8	93.6	2.5	95.0	1.3	+
8	5	151.0 (5:1)	30	7.2	92.0	2.5	95.0	1.3	+
9	5	30.0 (1:1)	90	7.5	91.7	2.9	87.4	0.9	+
10	5	151.0 (5:1)	90	6.4	92.9	2.7	92.1	1.1	+

Abbreviations: AE, aerobic endospores; CFU, colony‐forming unit; FBW, filter backwash water; ID, identification; rem., removal; Turb., turbidity; +, presence of peroxide residual; −, not applicable, not analyzed, or not detected.

*Note:* B1 and B2 are analytical blanks.

^a^ No pH changes.

**FIGURE 5:**
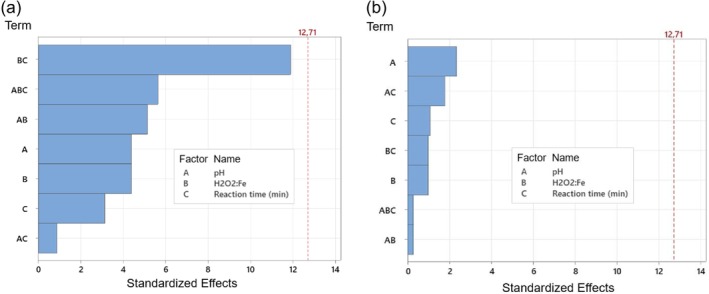
Pareto chart of the standardized effects, factorial experimental design 1 (FED1): (a) response is turbidity (NTU); *α* = 0.05; (b) response is AE removal efficiency (%); *α* = 0.05.

According to the Pareto chart analysis (Figure 5), no factor (pH; H_2_O_2_:Fe ratio; reaction time) or combination of factors presented significant influence (*α* = 0.05) on the removals of turbidity and AE from FBW. None of the conditions tested in the combined Fenton and sedimentation treatment achieved 2.0 log_10_ AE removal or final turbidity below 2.0 NTU, values adopted in this study as reference targets for evaluating the efficiency of FBW treatment (USEPA 2002). In accordance with the Filter Backwash Recycling Rule (FBRR), the recycling of FBW is permitted provided that it does not compromise the overall performance of the DWTP in achieving at least 2.0 log_10_ removal of *Cryptosporidium*. In California, it is further recommended that the turbidity of the recycling FBW be less than 2.0 NTU or, at most, correspond to half the turbidity of the raw water (USEPA 2002). In this context, supplementary treatment of FBW prior to recycling may serve as an additional barrier to enhance overall plant performance.

The test served as a preliminary basis for investigating optimal conditions. The results found at the central point, highlighting test ID 6, stood out for their proximity to the results of tests ID 7 and 8 in relation to AE removal. In addition, better performance was observed in turbidity reduction. Thus, it can be inferred that investigating operating conditions of 60 min reaction time, pH values between 4 and 5, and greater variation in the H_2_O_2_:Fe ratio may lead to better results.

### 3.3 Experimental Design 2

The results obtained from the 2^2^ factorial experimental design 2 (FED2) are presented in Table 4. The results showed that Fenton treatment achieved turbidity removals ranging from 97.6% to 98.9%. Test ID 1 and B2 achieved the lowest final turbidity values, 1.0 NTU and 0.8 NTU, respectively. To ensure better turbidity removal results, it may be necessary to optimize the H_2_O_2_:Fe ratio, in addition to operational adjustments in sedimentation time and pH.

**TABLE 4:** Concentrations and removal rates of turbidity, AE and EC obtained from the 2^2^ Factorial Experimental Design 2 (FED2): pH and initial H_2_O_2_ (mg·L^−1^) as factors.

Test ID	pH	Initial H_2_O_2_ (mg. L^−1^)	Turb.	Turb.	AE	AE	EC	EC rem. (%)	Peroxide residual
			(NTU)	rem. (%)	(log_10_ cfu. 100 mL^−1^)	rem. (log)	(log_10_ MPN. 100 mL^−1^)		
Raw FBW	7	−	90	−	4.0	−	2.3	−	−
B1	7^a^	−	2.5	97.2	2.5	1.5	1.0	95.0	−
B2	4	−	0.8	99.1	2.6	1.4	1.1	93.7	−
B3	5	−	2.2	97.6	2.4	1.6	0.9	96.0	−
1	4	31.0 (1:1)	1.0	98.9	1.7	2.3	< LOD		+
2	4	309.0 (10:1)	1.8	98.0	1.3	2.7	< LOD		+
3	4.5	93.0 (5.5:1)	1.6	98.2	1.9	2.1	< LOD		+
4	4.5	93.0 (5.5:1)	2.2	97.6	1.8	2.2	< LOD		+
5	5	31.0 (1:1)	2.0	97.8	2.5	1.5	0.5	98.4	+
6	5	309.0 (10:1)	2.2	97.6	2.5	1.5	< LOD		+

Abbreviations: AE, aerobic endospores; CFU, colony‐forming unit; EC, *Escherichia coli*; FBW, filter backwash water; ID, identification; LOD, limit of detection (1.0 MPN/100 mL); MPN, most probable number; rem., removal; Turb., turbidity; +, presence of peroxide residual; −, not applicable, not analyzed, or not detected.

*Note:* B1, B2, and B3 are analytical blanks.

^a^ No pH changes.

With regard to AE removal, reductions between 1.5 and 2.7 log_10_ were observed in the samples that underwent the Fenton reaction, with test ID 2 showing the best result (2.7 log_10_), as this test was performed at a lower pH and with the highest H_2_O_2_:Fe ratio (Table 4). Regarding EC removal, it was observed that, except for test ID 5, all other samples that underwent the Fenton reaction showed high removal efficiencies (≥ 98.4%), with final EC concentrations below the detection limit.

**FIGURE 6:**
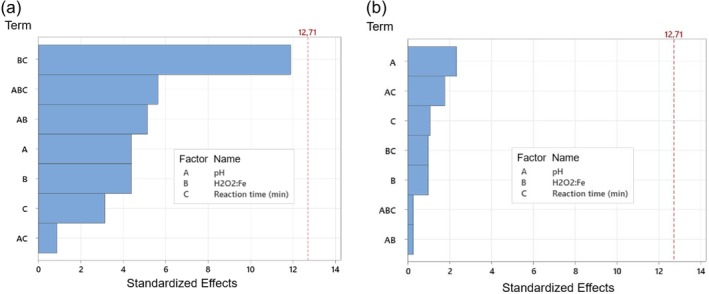
Pareto chart of the standardized effects, factorial experimental Design 2 (FED2): (a) response is turbidity (NTU); *α* = 0.05; (b) response is AE removal efficiency (%); *α* = 0.05.

**FIGURE 7:**
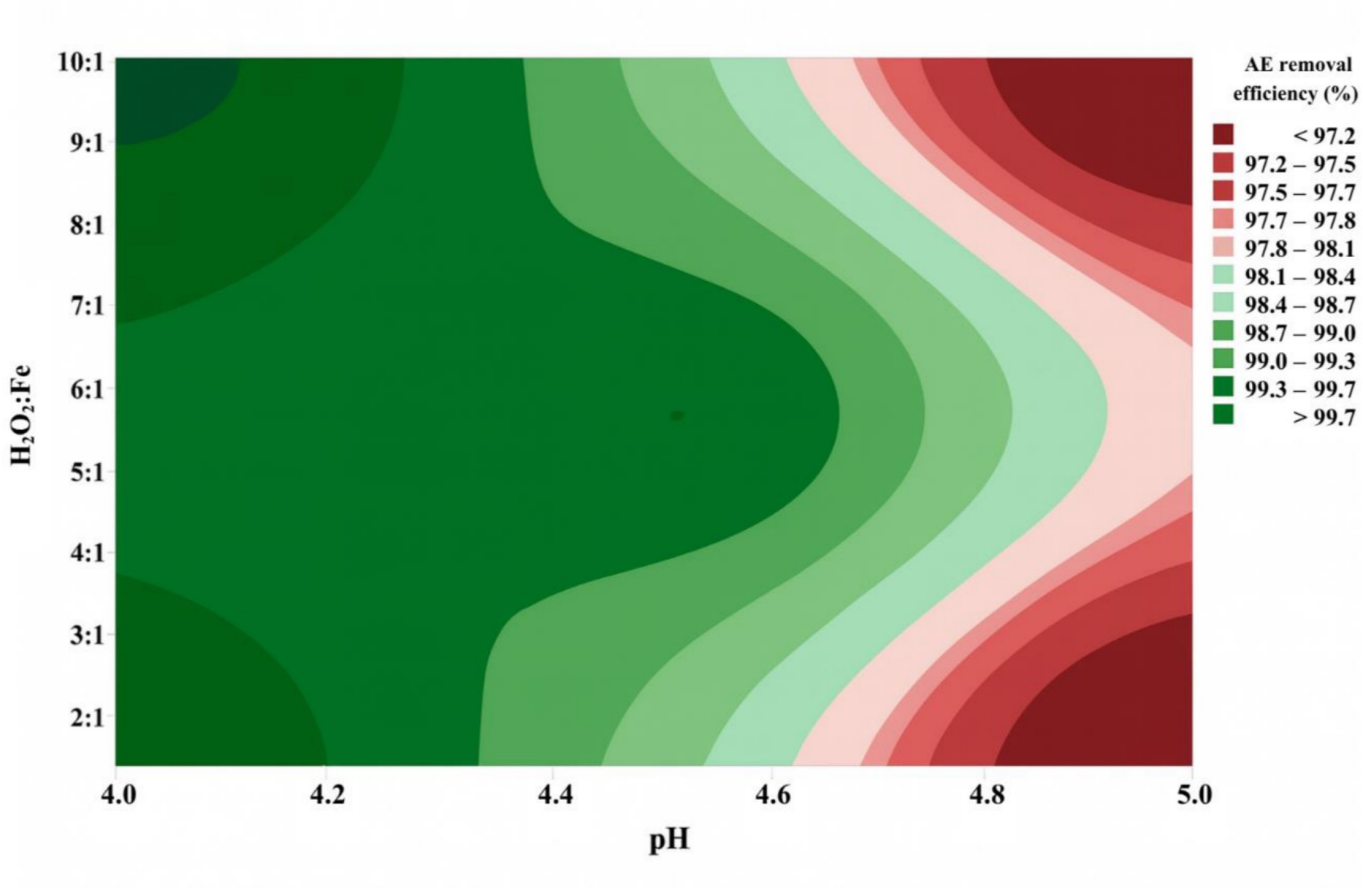
Contour plot of the second factorial experimental design 2 (FED2), concerning aerobic endospores (AE) removal efficiency (%).

Analyzing the Pareto chart of the data obtained for the turbidity response variable (Figure 6a), it cannot be stated that there was a significant difference at a significance level of 5%, but the condition of the experiment can be considered satisfactory because it achieved the turbidity values required for FBW recycling according to the FBRR (USEPA 2002). Regarding the removal of AE (Figure 6b), it was observed that pH had a significant influence on the result (*p*‐value = 0.032). It corroborates the fact that pH is a strong factor for AE removal, as tests showed the removal of at least 2.0 log_10_ of AE for recycling. Finally, regarding AE removals, analyzing the contour plot presented in Figure 7, it can be seen that an increase in pH decreases the efficiency of the process, whereas an increase in H_2_O_2_:Fe ratio has little influence on AE removal.

Arora et al. (2001) demonstrated that, under conventional clarification of FBW, *Cryptosporidium* oocysts were removed by 1.4 log_10_, whereas AE (*Bacillus*) exhibited markedly lower reductions of only 0.4 log_10_. This indicates greater persistence and resistance of bacterial spores compared to protozoan pathogens. In that study, microbial removal during clarification was not attributed to free settling of microorganisms, but rather to their association with suspended solids and flocs. The authors further noted that the presence of solids in FBW enhanced microbial removal, suggesting that protozoa and spores are predominantly removed through particle‐associated sedimentation mechanisms (Arora et al. 2001).

In the study by Matavos‐Aramyan et al. (2017), the efficacy of the Fenton process in reducing the viability of *Cryptosporidium parvum* oocysts was demonstrated, achieving approximately 52% (0.32 log_10_ removal) inactivation under the classical Fenton system at a high hydrogen peroxide concentration of 90 mmol (3060 mg·L^−1^) and a reaction time of only 2 min. The experiments were performed in water samples within a near‐neutral pH range (6–8), artificially inoculated with *C. parvum* oocysts, using ferrous sulphate as an external iron source, with Fe^2+^ concentrations of 80 mg·L^−1^ (Matavos‐Aramyan et al. 2017). These experimental conditions differ substantially from those typically observed in FBW, which is characterized by high turbidity, greater physicochemical complexity, and residual iron from the coagulation process. In contrast, in the present study, the reaction was conducted under acidic conditions and with considerably longer reaction times.

Although AE have been proposed as conservative surrogates for protozoan pathogens, direct validation of their behavior under Fenton‐based treatment remains limited. The susceptibility of highly resistant microbial structures such as AE to oxidative treatments may be considerably lower than that observed for protozoan (oo)cysts. Hydrogen peroxide is a useful antiseptic, being bactericidal at low concentrations, but only slowly sporicidal (Russell 1990), reflecting the remarkable resistance of bacterial spores to chemical oxidants. In this sense, oxidizing agents appear to exert their lethal action mainly by damaging the spore's inner membrane, perhaps through lipid peroxidation (Setlow 2006), rather than promoting rapid inactivation. Thus, the 2.7 log_10_ AE removal achieved in this study demonstrates a conservative potential to remove *Cryptosporidium* oocysts from FBW.

### 3.4 Final Fenton Treatment

Considering the results obtained from the FED2, a final experiment was conducted in triplicate using the optimal operational parameters (pH = 4; H_2_O_2_:Fe ratio = 10:1; reaction time = 60 min), and the results are presented in Table 5. Control tests without the addition of H_2_O_2_ were also performed. It is worth emphasizing that during the experiment there was no considerable variation in pH, with an average value of 3.96 ± 0.02 at 25 ± 1°C.

**TABLE 5:** Average results of optimal Fenton treatment in triplicate: pH = 4; H_2_O_2_:Fe ratio = 10:1; reaction time = 60 min.

Time	Initial H_2_O_2_ (mg. L^−1^)	Turbidity (NTU)^a^	AE (log_10_ cfu. 100 mL^−1^)	EC (log_10_ MPN. 100 mL^−1^)	Peroxide residual (mg·L^−1^)
T_0_ (before stirring)	−	66.1 ± 3.8	3.8	2.2	−
T_r_ (after stirring)	N/A	−	3.6	1.7	−
	119 (10:1)	−	3.6	< LOD	−
T_s_ (after sedimentation)	N/A	0.45 ± 0.02	1.2	< LOD	+
	119 (10:1)	0.60 ± 0.06	1.3	< LOD	+

Abbreviations: AE, aerobic endospores; CFU, colony‐forming unit; EC, *E. coli*; ID, identification; LOD, limit of detection (1.0 MPN/100 mL); MPN, most probable number; N/A, samples without H_2_O_2_ addition; +, presence of peroxide residual; −, not applicable, not analyzed, or not detected.

*Note:* T0: initial time, before the reaction; T_0_ = minute zero; T_r_ = reaction time (60 min); T_s_ = sedimentation time (120 min).

^a^ Mean ± standard deviation.

A reduction of more than 99% in turbidity was observed in the control tests (without H_2_O_2_ addition) and the Fenton reaction, with final average values of 0.45 NTU and 0.60 NTU, respectively, suggesting that pH adjustment alone may have contributed substantially to particle destabilization and settling, while the Fenton reaction provided comparable clarification performance. The turbidity reductions obtained in this study are consistent with the variability reported in the literature for sedimentation‐based treatment of FBW. Among the studies that evaluated sedimentation without coagulation/flocculation, turbidity values exceeding the 2.0 NTU benchmark have been reported in some cases, indicating that settling alone may not always ensure high clarification efficiency (Adin et al. 2002; Raj et al. 2008; Yang et al. 2006). Conversely, high‐performance outcomes have also been documented, such as a final turbidity of 0.9 NTU after 9 h of settling (Arendze and Sibiya 2014) and approximately 1.0 NTU after 2 h in full‐scale DWTP in the USA (Tobiason et al. 2003).

In addition, complete EC inactivation was observed after 60 min of the Fenton reaction, which did not occur in the control tests, demonstrating the efficiency of the AOP/Fenton treatment at removing bacterial indicators. On the other hand, AE oxidation resulted in low removals: 0.19 log_10_ in the control tests and 0.17 log_10_ in the Fenton treatment. After sedimentation, removals were greater than 2.0 log_10_ in all cases.

The results suggest that AE removal occurs through adsorption of microorganisms to suspended particles and then sedimentation. However, the continuity of the Fenton reaction cannot be ruled out even after the end of agitation, since at the end of sedimentation it was still possible to detect the oxidant in the supernatant. Therefore, it is recommended to investigate the Fenton reaction over longer periods and to quantify AE in the final sludge.

In addition, it is important to note that the total iron concentration in the raw sample in the final treatment experiment (18 mg·L^−1^) was considerably lower than the other two experiments performed (44 mg·L^−1^; FED1 and FED2). This result reinforces the relevance of investigating the minimum iron concentration necessary to improve AE removal in Fenton processes, considering the potential use of AE as an indicator of the removal of *Giardia* sp. cysts and *Cryptosporidium* sp. oocysts. The difference in iron concentration may have played a crucial role in the Fenton reaction, potentially influencing the results.

The presence of residual peroxide, especially in samples with higher amounts of added peroxide, indicates that H_2_O_2_ is not being fully consumed in the reaction, which may be related to the low iron concentration or short reaction monitoring time.

The effectiveness of the Fenton process does not follow a simple linear relationship with pH or hydrogen peroxide concentration. This limitation suggests the possibility of an influence on iron concentration. Therefore, this additional factor highlights the importance of understanding the complexity of interactions in samples to obtain a more accurate treatment result. This process may require further investigation of the interactions between variables, considering the sample factors that influence the results.

## 4 Conclusions

This work showed the application of AOP/Fenton treatment of FBW using the iron already present in the matrix. The results indicate that this is operationally feasible and effective aiming for FBW recycling in DWTP. At bench scale, EC was fully inactivated (not detected after a 60‐min reaction), and turbidity removals were ≥ 99%, resulting in final turbidity < 1.0 NTU after sedimentation under optimal operational parameters (pH ≈ 4; H_2_O_2_:Fe = 10:1). For AE, a surrogate for protozoa (oo)cysts, oxidation alone provided limited removal during the Fenton reaction alone. However, by combining the Fenton reaction with a sedimentation step, ≥ 2.0 log_10_ reductions were observed, fulfilling the safety criterion for water recycling. Factorial analysis identified pH as the key driver for AE removal, whereas increasing the H_2_O_2_:Fe ratio resulted in only marginal benefits. Variations in total iron (18 vs. 44 mg·L^−1^) likely affected the AOP/Fenton performance, highlighting the need to establish the minimum effective Fe concentration and refine the optimal operating parameters in terms of pH, H_2_O_2_:Fe ratio, and reaction/sedimentation times. Notably, the lowest turbidity values were sometimes observed without the addition of H_2_O_2_, suggesting that clarification through coagulation/adsorption and settling primarily governs the removal of suspended solids and AE, whereas the AOP/Fenton process is effective and decisive for EC inactivation.

## Author Contributions

**Luana Valverde Pereira:** formal analysis, data curation, software, visualization, writing – original draft, writing – review and editing. **Matheus Carlos de Sá:** formal analysis, data curation, software, visualization, writing – original draft, writing – review and editing. **Isabella Pereira de Lima:** formal analysis. **Lucas Coelho Rossi:** formal analysis. **Taciane de Oliveira Gomes de Assunção:** formal analysis, data curation, writing – review and editing. **Edgard Henrique Oliveira Dias:** methodology, writing – review and editing. **Sue Ellen Costa Bottrel:** conceptualization, funding acquisition, project administration, methodology, writing – original draft, writing – review and editing. **Renata de Oliveira Pereira:** conceptualization, funding acquisition, project administration, methodology, writing – original draft, writing – review and editing.

## Funding

The authors acknowledge the financial support from the Minas Gerais State Agency for Research and Development (FAPEMIG) grant APQ‐01329‐22.

## Ethics Statement

The authors have nothing to report.

## Conflicts of Interest

The authors declare no conflicts of interest.

## Data Availability

Data will be made available on request.
